# Predicting 1-year non-cancer-related adverse events after lung resection

**DOI:** 10.1093/icvts/ivad199

**Published:** 2023-12-12

**Authors:** Takashi Eguchi, Shogo Ide, Shunichiro Matsuoka, Yasuhiro Iijima, Shuji Mishima, Daisuke Hara, Hirotaka Kumeda, Kentaro Miura, Kazutoshi Hamanaka, Kimihiro Shimizu

**Affiliations:** Division of General Thoracic Surgery, Department of Surgery, Shinshu University School of Medicine, Matsumoto, Japan; Division of General Thoracic Surgery, Department of Surgery, Shinshu University School of Medicine, Matsumoto, Japan; Division of General Thoracic Surgery, Department of Surgery, Shinshu University School of Medicine, Matsumoto, Japan; Division of General Thoracic Surgery, Department of Surgery, Shinshu University School of Medicine, Matsumoto, Japan; Division of General Thoracic Surgery, Department of Surgery, Shinshu University School of Medicine, Matsumoto, Japan; Division of General Thoracic Surgery, Department of Surgery, Shinshu University School of Medicine, Matsumoto, Japan; Division of General Thoracic Surgery, Department of Surgery, Shinshu University School of Medicine, Matsumoto, Japan; Division of General Thoracic Surgery, Department of Surgery, Shinshu University School of Medicine, Matsumoto, Japan; Division of General Thoracic Surgery, Department of Surgery, Shinshu University School of Medicine, Matsumoto, Japan; Division of General Thoracic Surgery, Department of Surgery, Shinshu University School of Medicine, Matsumoto, Japan

**Keywords:** Anatomical lung resection, Atrial fibrillation, Nomogram, Non-cancer-related outcome, Resected volume, Segmentectomy

## Abstract

**OBJECTIVES:**

Assessing the risk for non-cancer-related outcomes following lung cancer surgery is crucial for high-risk patients. This study examined non-cancer-related adverse events within 1 year after lung resection, emphasizing the role of resected lung volume and postoperative atrial fibrillation (POAF).

**METHODS:**

We conducted a retrospective analysis of 460 patients who underwent anatomical lung resection for malignant lung tumours. We assessed perioperative factors, such as the number of resected subsegments and POAF, as potential predictors of 1-year non-cancer-related adverse events. Additionally, we validated a previously published nomogram for predicting POAF.

**RESULTS:**

One-year non-cancer-related adverse events occurred in 20% of patients. Multivariable analysis identified higher age, lower percentage-predicted forced expiratory volume in 1 second, greater number of resected subsegments and POAF as independent predictors of these adverse events. The incidence of POAF was 8.5%, with higher age, history of atrial fibrillation, and open thoracotomy as independent predictors. A temporal link between POAF and other severe postoperative complications was observed, as 71% of POAF cases preceded other complications. The nomogram's predicted risk for POAF was associated well with the actual incidence.

**CONCLUSIONS:**

Resected lung volume and POAF are statistically significant factors associated with non-cancer-related outcomes after lung resection. Minimizing resected lung volume when oncologically and technically feasible, along with identifying patients at risk for POAF, may contribute to improved postoperative outcomes. Our results have implications for risk stratification and preoperative decision-making in lung cancer surgery.

**Figure:**
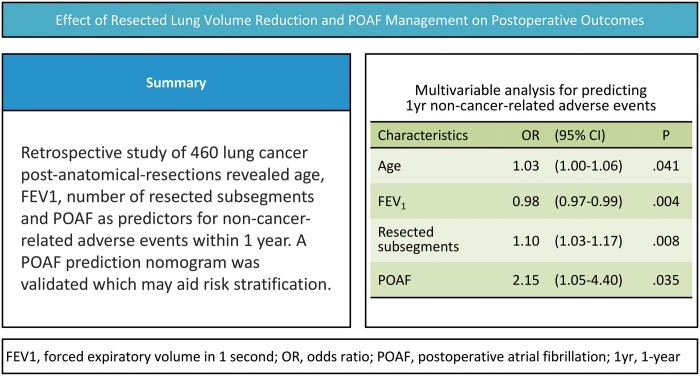

## INTRODUCTION

The rise in early-stage lung cancer detection in an ageing society has led to an increase in lung resections performed on high-risk patients. It is essential to accurately assess non-cancer-related postoperative outcomes for these patients, including the elderly and those with multiple comorbidities [1].

Past research on cause-specific mortality following lobectomy for early-stage lung cancer has revealed a higher incidence of non-cancer-related deaths compared to lung cancer-related deaths up to 1.5 years post-surgery in elderly patients [1]. Moreover, early-phase non-cancer-specific death was strongly associated with severe postoperative morbidity [1, 2]. A large prospective randomized trial (JCOG0802/WJOG4607L) comparing segmentectomy and lobectomy outcomes for small lung cancers found a higher occurrence of deaths from respiratory disease, cerebrovascular disease, and cancers other than lung cancer in the lobectomy group compared to that in the segmentectomy group [3]. This disparity in non-lung-cancer-related deaths between the 2 procedures suggests that increased resected lung volume may contribute to non-cancer-related adverse events in the early postoperative phase. While some studies have investigated postoperative pulmonary function [4, 5], none have explored the relationship between resected lung volume and early-phase non-cancer-related outcomes.

Postoperative atrial fibrillation (POAF) is one of the most frequent adverse events following lung resection [6] and is linked to frequent relapse, cerebrovascular thrombotic events, and bleeding events due to anticoagulation therapy [7]. Research also suggests that POAF may precede other severe adverse events [8]. Given the previously reported link between higher POAF risk and larger extent of lung resection [9], we hypothesized a potential connection between POAF, resected volume, and non-cancer-related adverse events. Understanding this relationship is important as it may impact the risk assessment and management of high-risk patients undergoing lung resection. Thus, predicting POAF is vital for thoracic surgery in high-risk patients. A brain natriuretic peptide (BNP) -based prediction model for POAF has been proposed [9], but its applicability to the global population remains uncertain.

This study aimed to investigate the risk for non-cancer-related adverse events within 1 year after lung resection, with a focus on resected lung volume and POAF, to improve non-cancer-related postoperative outcomes in high-risk patients. The number of resected subsegments was used as a surrogate marker of resected lung volume. Additionally, the potential temporal relationship between POAF and other complications was evaluated, and a previously published POAF prediction model was validated using our patient cohort. Our study focuses on the investigation of non-cancer-related adverse events following lung resection with curative intent. While we recognize the inherent risks associated with surgical intervention, our aim is not to compare the risks of surgery and cancer but to understand the factors contributing to adverse events unrelated to cancer, which may help in optimizing patient care and outcomes.

## PATIENTS AND METHODS

### Ethical statement

We confirm that all research reported in this paper that involves human participants was conducted in accordance with the universally accepted principles of the Declaration of Helsinki. An opt-out approach was adopted instead of obtaining written informed consent. The approval was obtained from the institutional review board of Shinshu University Hospital (Project ID 5498, approved on 13 April 2022).

### Study cohort

**Figure 1:**
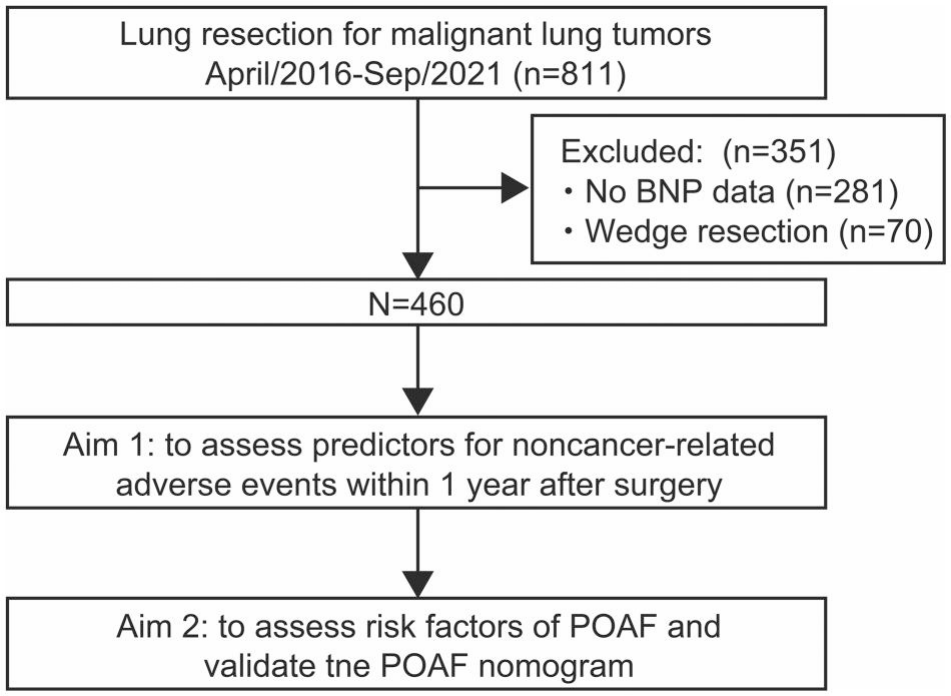
A schema of the study cohort. BNP: brain natriuretic peptide; POAF: postoperative atrial fibrillation.

The present study was a single-institution retrospective study approved by the institutional review board of Shinshu University Hospital (Project ID 5498). An opt-out approach was utilized instead of obtaining written informed consent from each patient. We investigated a total of 811 consecutive patients who underwent curative-intent lung resection for primary and metastatic lung tumours between 1 April 2016 and 31 September 2021, at Shinshu University Hospital, Matsumoto, Japan. After excluding 281 patients without sufficient data collection and 70 patients who underwent wedge resection, we analysed the remaining 460 patients in our study cohort (Fig. 1). Most thoracotomies during the study were either anterolateral or posterolateral depending on the stage of the disease.

### Clinicopathological data collection

Patient demographic information was obtained from the prospectively maintained thoracic surgery database of the Division of Thoracic Surgery at Shinshu University Hospital. The data on clinicopathological variables were obtained by reviewing patient medical records specifically for this study. All preoperative variables were evaluated within 3 months prior to surgery.

### One-year non-cancer-related adverse events

The primary outcome of this study was 1-year non-cancer-related adverse events. These were defined as any severe morbidities and mortalities, graded at or above level 3 according to the Common Terminology Criteria for Adverse Events, occurring within 1 year after surgery. This includes both postoperative complications directly related to surgical interventions and other events not directly related to the interventions. Any deaths directly related to malignant diseases for which a curative-intent lung resection was performed during the study period, including lung cancer deaths in patients with primary lung cancer and other cancer deaths in patients with metastatic lung cancer, were regarded as cancer-related deaths and excluded from non-cancer-related adverse events in this study. Deaths related to other malignancies that were not treated by lung resection in this study were regarded as non-cancer-related deaths and were included in non-cancer-related adverse events.

### Postoperative atrial fibrillation

We evaluated clinical factors associated with POAF. In this study, POAF was defined as the occurrence of atrial fibrillation within 30 days after surgery, regardless of its grade and duration. We also validated the predictive value for POAF of a previously published BNP-based model by Amar *et al.* [9] using data obtained from our study cohort.

### Number of resected subsegments

We used the number of resected subsegments as a surrogate for resected lung volume. The right lung has 22 subsegments: 6 in the upper lobe, 4 in the middle and 12 in the lower. The left lung has 20: 10 in the upper and 10 in the lower [5]. In cases of extended segmentectomy or additional wedge resection, we added 0.5 to the count.

### Primary analysis

The primary analysis investigated non-cancer-related adverse events within the first year after lung resection, focusing on severe morbidities and mortalities graded at or above level 3 according to the Common Terminology Criteria for Adverse Events. We aimed to identify key factors influencing these events and to understand their impact on patient outcomes.

### Sub-analyses

We conducted sub-analyses to explore specific aspects of non-cancer-related adverse events:

1. Elderly and younger patients were compared to analyse age-related variations in outcomes.
2. POAF was investigated as a potential precursor for other complications.
3. A previously published model for predicting POAF was validated using our cohort.

These sub-analyses aimed to provide a comprehensive understanding of the factors influencing non-cancer-related adverse events and to validate existing predictive tools.

### Statistical methods

The data are presented as number (percentage) or median (interquartile range). The association between factors was evaluated using Mann–Whitney *U* test for continuous variables and Fisher's exact test for categorical variables. In the logistic regression analysis, we performed standard diagnostics to assess the fit and assumptions of the model. The diagnostics included checks for influence measures, multicollinearity, and receiver-operating characteristic curve analysis. Age was categorized into groups to investigate specific effects within different age groups. A multivariable binary logistic regression analysis was conducted to determine the independent predictors for outcomes. Any factor with a *P*-value of ≤0.1 in the univariable analysis was included in the multivariable analysis. Variable selection was performed using a backward selection strategy based on p-values. The model assumptions in multivariable logistic regression analyses were checked by examining the relationships between the predictors and the outcome. This included assessing linearity, checking for multicollinearity, and ensuring that the error terms were independently and identically distributed. We performed all statistical analyses using IBM SPSS Statistics version 27 (IBM, Armonk, NY, USA) software, and statistical significance was set at *P* < 0.05.

## RESULTS

### Patient demographics and 1-year non-cancer-related adverse events

Table 1 presents the demographics of 460 patients and a comparison between those who experienced 1-year non-cancer-related adverse events and those who did not. Among the 460 patients, 298 underwent lobectomy and 162 underwent segmentectomy, with a median of 6 resected subsegments. Of the total patients, 94 (20%) patients experienced non-cancer-related adverse events within 1 year, and Supplementary Material, Table S1 provides a breakdown of these events. Of the 14 deaths in the cohort, 11 were non-cancer-related, 2 were lung cancer-related and 1 was related to primary soft tissue malignancy. The majority of severe morbidities (*n* = 83) were respiratory diseases (*n* = 66, 80%), followed by cardiovascular diseases (*n* = 14, 17%).

**Table 1:** Patient demographics and comparison between patients with and without non-cancer-related adverse events within 1 year

Characteristics	All cases (*n* = 460)		Non-cancer-related adverse events within 1 year				*P-*value
			No (*n* = 366) (84%)^a^		Yes (*n* = 94) (16%)^a^		
Age (years)	71 (64–76)		70 (63–46)		74 (69–78)		**0.003**
Sex							
Male	248 (54)		183 (50)		65 (69)		**0.001**
Female	212 (46)		183 (50)		29 (31)		
BMI (kg/m^2^)	23 (20–25)		23 (21–25)		22 (20–25)		0.27
Smoking history							
Never	205 (44)		172 (47)		33 (35)		**0.048**
Former	242 (53)		186 (51)		56 (60)		
Current	13 (3)		8 (2)		5 (5)		
Hypertension							
No	241 (52)		191 (52)		50 (53)		0.86
Yes	219 (48)		175 (48)		44 (47)		
Coronary artery disease							
No	418 (91)		338 (92)		80 (85)		**0.030**
Yes	42 (9)		28 (8)		14 (15)		
Diabetes mellitus							
No	365 (79)		291 (80)		74 (79)		0.87
Yes	95 (21)		75 (20)		20 (21)		
History of atrial fibrillation							
No	433 (94)		346 (95)		87 (93)		0.47
Yes	27 (6)		20 (5)		7 (7)		
Chemotherapy							
No	448 (97)		357 (98)		91 (97)		0.69
Yes	12 (3)		9 (2)		3 (3)		
BNP (pg/ml)	23 (13–41)		22 (12–38)		31 (17–56)		**0.002**
%FEV_1_ (%)	99 (86–113)		100 (88–113)		90 (77–110)		**0.002**
Surgical approach							
MIS	382 (83)		312 (85)		70 (75)		**0.013**
Thoracotomy	78 (17)		54 (15)		24 (25)		
Surgery							
Segmentectomy	162 (35)		135 (37)		27 (29)		0.14
Lobectomy	298 (65)		231 (63)		67 (71)		
Number of resected subsegments	6 (4–10)		6 (4–10)		10 (6–10)		**0.003**
POAF							
No	421 (92)		342 (93)		79 (84)		**0.004**
Yes	39 (8)		24 (7)		15 (16)		

Data are shown as number (%) or median (25–75 percentiles).

a Percentages among all 460 patients.

BMI: body mass index; BNP: brain natriuretic peptide; %FEV_1_: percentage-predicted forced expiratory volume in 1 s; MIS: minimally invasive surgery; POAF: postoperative atrial fibrillation.

The bold font indicates statistical significance.

The results of a univariable logistic regression analysis showed that a higher age, male sex, smoking history, history of coronary disease, higher BNP, lower percentage-predicted forced expiratory volume in 1 s, open thoracotomy, a larger number of resected subsegments and POAF were significantly associated with a higher risk for 1-year non-cancer-related adverse events (Table 2). On multivariable analysis, higher age, lower percentage-predicted forced expiratory volume in 1 s, a larger number of resected subsegments and POAF were identified as independent predictors of 1-year non-cancer-related adverse events (Table 2).

**Table 2:** Logistic regression analysis for predicting non-cancer-related adverse events within 1 year

Characteristics	Univariable			Multivariable^a^		
	OR	(95% CI)	*P-*value	OR	(95% CI)	*P-*value
Age (per 1 year increase)	1.02	(0.99–1.05)	0.062	1.03	(1.00-1.06)	**0.041**
Sex female (versus male)	0.45	(0.28–0.72)	**0.001**			
BMI (per 1 kg/m^2^ increase)	0.95	(0.88–1.02)	0.12			
Smoking history						
Never	1		0.054			
Former	1.57	(0.97–2.53)				
Current	3.26	(1.00–10.59)				
Hypertension (versus none)	0.96	(0.61–1.51)	0.86			
Coronary artery disease (versus none)	2.11	(1.06–4.20)	**0.033**			
Diabetes mellitus (versus none)	0.05	(0.60–1.83)	0.87			
History of atrial fibrillation (versus none)	1.39	(0.57–3.40)	0.47			
Chemotherapy (versus none)	1.31	(0.35–4.93)	0.69			
BNP (per 1 pg/ml increase)	1.01	(1.00–1.01)	**0.021**			
%FEV_1_ (per 1% increase)	0.98	(0.97–0.99)	**0.003**	0.98	(0.97-0.99)	**0.004**
Surgical approach MIS (versus thoracotomy)	0.51	(0.29–0.87)	**0.014**			
Lobectomy (versus segmentectomy)	1.45	(0.88–2.38)	0.14			
Number of resected subsegments (per 1 increase)	1.11	(1.04–1.18)	**0.002**	1.10	(1.03-1.17)	**0.008**
POAF (versus none)	2.71	(1.36–5.39)	**0.005**	2.15	(1.05-4.40)	**0.035**

a Multivariable analysis using a backward selection strategy, starting with factors with a *P*-value of ≤0.1 in univariable analysis.

BMI: body mass index; BNP: brain natriuretic peptide; CI: confidence interval; %FEV_1_: percentage-predicted forced expiratory volume in 1 s; MIS: minimally invasive surgery; OR: odds ratio; POAF: postoperative atrial fibrillation.

The bold font indicates statistical significance.

### Comparison between elderly (≥75 years) and younger (<75 years) patients

A comparison of grade distribution of 1-year non-cancer-related adverse events between elderly (≥75 years) and younger (<75 years) cohorts is shown in Supplementary Material, Table S2. The risk for 1-year non-cancer-related adverse events in the elderly cohort was significantly associated with higher grades than that in the younger cohort (*P* = 0.013).

In the logistic regression analysis to predict 1-year non-cancer-related adverse events only in elderly patients (≥75 years, *n* = 156), male sex, a larger number of resected subsegments, and POAF were independent predictors of 1-year non-cancer-related adverse events (Supplementary Material, Tables S3 and S4). Although we considered factors such as BNP and preoperative comorbidity, comprehensive information regarding the patients' preoperative cardiac condition is lacking. This shortcoming could potentially impact the interpretation of our findings.

### Temporal relationship between postoperative atrial fibrillation and severe postoperative complications: postoperative atrial fibrillation as a precursor for other complications

In this cohort, 39 patients (8%) experienced POAF within 30 days after surgery, and all POAF cases were of grade 2. Of these, 14 patients with POAF (36%) also experienced other severe postoperative complications during hospitalization (grade ≥3). Among these 14 patients, 10 (71%) patients experienced POAF before the onset of subsequent severe complications (9 respiratory and 1 cardiovascular), while the remaining 4 patients (29%) developed POAF either simultaneously with or after the other complications.

### Postoperative atrial fibrillation prediction

Table 3 shows a comparison between patients with POAF and those without. The univariable analysis revealed that POAF was significantly associated with higher age, history of hypertension, history of atrial fibrillation, higher BNP, open thoracotomy, lobectomy, and a larger number of resected subsegments (Table 4). The multivariable analysis indicated that higher age, history of atrial fibrillation, and open thoracotomy were independent predictors of POAF occurrence (Table 4). We incorporated an interaction term between the type of surgery (lobectomy or segmentectomy) and the number of resected subsegments to capture synergistic effects. This analysis displayed a trend towards significance, suggesting that the risk profile for POAF varies depending on both the surgery type and extent of resection (Table 4).

**Table 3:** Demographics of all patients and comparison with and without postoperative atrial fibrillation

Characteristics	All cases (*n* = 460)		POAF				*P-*value
			No (*n* = 421) (92%)^a^		Yes (*n* = 39) (8%)^a^		
Age (years)	71 (64–76)		71 (64–76)		74 (69–79)		**0.007**
Sex							
Male	248 (54)		222 (53)		26 (67)		0.095
Female	212 (46)		199 (47)		13 (33)		
BMI (kg/m^2^)	23 (20–25)		23 (20–25)		22 (21–23)		0.14
Smoking history							
Never	205 (44)		191 (45)		14 (36)		0.40
Former	242 (53)		219 (52)		23 (59)		
Current	13 (3)		11 (3)		2 (5)		
Hypertension							
No	241 (52)		227 (54)		14 (36)		**0.031**
Yes	219 (48)		194 (46)		25 (64)		
Coronary artery disease							
No	418 (91)		383 (91)		35 (90)		0.80
Yes	42 (9)		38 (9)		4 (10)		
Diabetes mellitus							
No	365 (79)		333 (79)		32 (82)		0.63
Yes	95 (21)		88 (21)		7 (18)		
History of atrial fibrillation							
No	433 (94)		400 (95)		33 (85)		0.008
Yes	27 (6)		21 (5)		6 (15)		
Chemotherapy							
No	448 (97)		410 (97)		38 (97)		0.99
Yes	12 (3)		11 (3)		1 (3)		
Preoperative β-blocker use							
No	427 (93)		393 (93)		34 (87)		0.15
Yes	33 (7)		28 (7)		5 (13)		
Preoperative calcium-channel blocker use							
No	304 (66)		227 (66)		27 (69)		0.70
Yes	155 (34)		143 (34)		12 (31)		
Preoperative heart rate (bpm)	69 (61–77)		69 (62–77)		69 (60–76)		0.69
BNP (pg/ml)	23 (13–41)		22 (12–39)		32 (19–74)		**0.002**
%FEV_1_ (%)	99 (86–113)		99 (86–113)		91 (79–107)		0.11
Surgical approach							
MIS	382 (83)		357 (85)		14 (36)		**0.001**
Thoracotomy	78 (17)		64 (15)		25 (64)		
Type of surgery							
Segmentectomy	162 (35)		155 (37)		7 (18)		**0.018**
Lobectomy	298 (65)		266 (63)		32 (82)		
Number of resected subsegments	6 (4–10)		6 (4–10)		10 (6–10)		**0.041**

Data are shown as number (%) or median (25–75 percentiles).

a Percentages among all 460 patients.

BMI: body mass index; BNP: brain natriuretic peptide; %FEV_1_: percentage-predicted forced expiratory volume in 1 s; MIS: minimally invasive surgery; POAF: postoperative atrial fibrillation.

The bold font indicates statistical significance.

**Table 4:** Logistic regression analysis for the probability of postoperative atrial fibrillation

Characteristics	Univariable			Multivariable^a^		
	OR	(95% CI)	*P-*value	OR	(95% CI)	*P-*value
Age (per 1 year increase)	1.06	(1.02–1.11)	**0.008**	1.06	(1.01–1.10)	**0.013**
Sex female (versus male)	0.56	(0.28–1.12)	0.099			
BMI (per 1 kg/m^2^ increase)	0.93	(0.83–1.03)	0.14			
Smoking history						
Never	1		0.41			
Former	1.43	(0.72–2.86)				
Current	2.48	(0.50–12.30)				
Hypertension (versus none)	2.09	(1.06–4.13)	**0.034**			
Coronary artery disease (versus none)	1.15	(0.34–3.42)	0.80			
Diabetes mellitus (versus none)	0.83	(0.35–1.94)	0.66			
History of atrial fibrillation (versus none)	3.46	(1.31–9.18)	**0.012**	3.02	(1.05–8.66)	**0.040**
Chemotherapy (versus none)	0.98	(0.12–7.80)	0.98			
Preoperative β-blocker use (versus none)	2.06	(0.75–5.69)	0.16			
Preoperative calcium-channel blocker use (versus none)	0.86	(0.42–1.75)	0.68			
Preoperative heart rate (per 1 bpm increase)	0.98	(0.96–1.01)	0.98			
BNP (per 1 pg/ml increase)	1.01	(1.00–1.01)	**0.013**			
%FEV_1_ (per 1 % increase)	0.99	(0.97–1.00)	0.14			
Surgical approach MIS (versus thoracotomy)	0.32	(0.16–0.65)	**0.002**	0.34	(0.16–0.74)	**0.006**
Type of surgery: lobectomy (versus segmentectomy)	2.66	(1.15–6.18)	**0.022**	8.94	(0.94–84.66)	0.056
Number of resected subsegments (per 1 increase)	1.10	(1.00–1.21)	**0.045**	2.69	(0.86–8.37)	0.088
Type of resection_number of resected subsegments^b^				0.72	(0.48–1.06)	0.093

a Multivariable analysis using a backward selection strategy, starting with factor with *P*-value ≤0.1 in univariable analysis.

b Interaction term between the type of surgery and the number of resected subsegments.

BMI: body mass index; BNP: brain natriuretic peptide; CI: confidence interval; %FEV_1_: percentage-predicted forced expiratory volume in 1 s; MIS: minimally invasive surgery; OR: odds ratio; POAF: postoperative atrial fibrillation.

The bold font indicates statistical significance.

### Validation of a previously published postoperative atrial fibrillation prediction model

**Figure 2:**
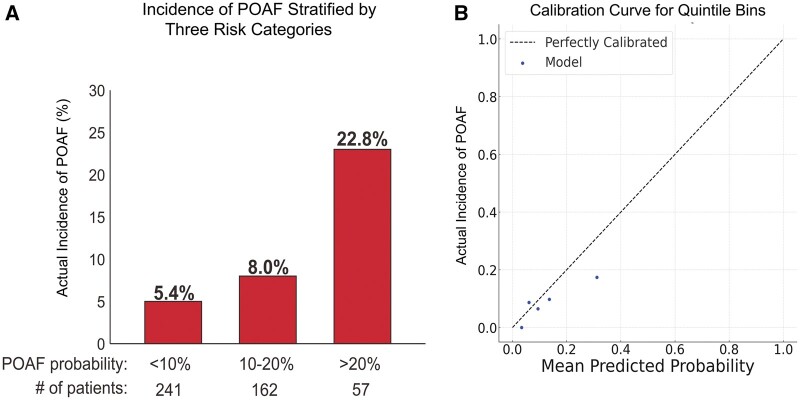
Validation for predicting POAF. (**A**) Incidence of POAF stratified by 3 risk categories. Patients were categorized into 3 risk groups based on their calculated POAF probabilities using the nomogram. The 95% confidence intervals for the actual incidence of POAF in these risk categories are as follows: for POAF probability <10%, 2.5–8.2%; for 10–20%, 3.8–12.2%; and for >20%, 11.9–33.7%. (**B**) Quintile-based calibration curve for POAF prediction. Patients were stratified into quintiles based on their predicted probability of experiencing POAF. The mean predicted probability of experiencing POAF within each quintile was plotted against the observed rate. The curve is an external validation metric and provided a more granular assessment of the model's predictive accuracy. POAF: postoperative atrial fibrillation.

We validated a nomogram proposed by Amar *et al.* [9] for predicting POAF after thoracic surgery. Patients were grouped into 3 categories based on the estimated risk for POAF (Fig. 2A); the incidence of POAF increased as the risk category became higher.

We further validated our model using a quintile-based calibration curve analysis (Fig. 2B). Our results showed that the model's predictions closely match the actual POAF rates, indicating good reliability. Although the model had some extreme predictions, overall, the quintile-based calibration curve supported its reliability in predicting POAF in our cohort.

## DISCUSSION

This study investigated factors associated with non-cancer-related adverse events within 1 year after lung resection, with a focus on early-phase complications in the risk assessment for non-cancer outcomes in high-risk patients, including the elderly. The strengths and novelty of this study include: (i) the emphasis on early-phase non-cancer-related adverse events and their assessment in high-risk patients; (ii) the identification of POAF as an independent risk factor of non-cancer-related adverse events, and validation of a published nomogram that can predict POAF preoperatively, leading to better treatment decisions and perioperative care for high-risk patients; and (iii) the first demonstration of a statistically significant association between the resected number of subsegments and non-cancer-related outcomes, suggesting that reducing resected lung volume could improve non-cancer-related outcomes beyond pulmonary function test values. The primary aim of our surgical approach was curative-intent lung resection, with the ultimate goal of achieving a tumour-free status. While we recognize the significance of non-cancer-related outcomes such as POAF, our study does not suggest reducing the number of resected subsegments solely for this purpose. Rather, we advocate for a patient-centred approach, where the volume of resected lungs may be reduced if it is oncologically and technically feasible, considering individual patient conditions and overall treatment goals.

Our study identified a strong link between the number of subsegments removed during surgery and the likelihood of experiencing non-cancer-related adverse events within a year after the procedure. Although we did not assess postoperative pulmonary function or long-term survival, our results suggest that lung-preserving procedures, including segmentectomy, could have positive effects on non-cancer-related outcomes compared to lobectomy, consistent with the findings of the JCOG0802 trial [3]. The favourable short-term non-cancer-related mortality after sublobar resection, compared to that after lobectomy, in the CALGB140503 trial (30- and 90-day mortality rates of 0.6% and 1.2% after sublobar resection and 1.1% and 1.7% after lobectomy) further supports these findings [10, 11]. Additional subgroup analyses of those trials may provide more insights into the relationship between resected lung volume and non-cancer-related outcomes.

Our study, along with previous reports, aimed to assess the risk for POAF after lung resection and its potential relationship with other non-cancer-related outcomes [8, 9]. Roselli *et al.* found that one-third of patients with POAF experienced other complications, and POAF often preceded these, particularly respiratory and infectious complications. This suggests that hypoxic and septic processes might trigger POAF [8]. Similarly, in our study, 36% of POAFs were followed by severe complications, mostly respiratory (90%), with POAF preceding in 71% of cases. Our findings support the hypothesis that POAF may be a precursor of hypoxic and septic processes after lung resection, but further investigations are needed to validate this hypothesis.

Given the statistically significant link between POAF and non-cancer-related outcomes, predicting POAF in high-risk patients is crucial for safe thoracic surgery. Our study successfully validated a previously published POAF prediction model, suggesting that the model may help improve perioperative patient care after lung resection and can be potentially applied across different regions worldwide.

### Limitations

This study has several limitations. First, it is a retrospective, single-institution study with a small cohort, and 281 patients were excluded due to incomplete data. Second, POAF detection was limited to the hospitalization period; thus, the analysis may have potentially missed post-discharge events. Third, the non-cancer-related adverse events include conditions such as prolonged air leakage and pleural effusion, which might have only influenced the duration of hospitalization. However, they may also have long-term functional implications, such as the need for pleurodesis. Therefore, we have chosen to include them as adverse events in the study. Fourth, our study did not account for any specific anatomical variations during lung resections, such as the precise location of venous ligation at the hilum. The ligation of the segmental vein is distant from the ostium of the homolateral lobar pulmonary vein, which could have notable implications for the AF triggering zone. While our multivariable analysis did show a trend towards the significance of lobectomy as a risk factor for POAF, it is important to note that we did not examine other potentially impactful variables. These include the location of venous ligation, the number of veins divided, and the length of the venous stump. Such factors may influence the occurrence of POAF and its analysis could provide additional insights not captured in our current analysis. Fifth, we did not evaluate lung diffusion capacity or postoperative pulmonary function, both of which could be significant predictors of non-cancer-related outcomes [1, 2]. Sixth, the study did not analyse the impact of postoperative pain management methods on POAF. Finally, our study found an association between POAF and respiratory issues, but we could not establish a direct cause-and-effect relationship due to limitations of our retrospective study design. Future research should address these limitations for a more comprehensive understanding of the relationship between non-cancer-related adverse events and lung resection.

## CONCLUSION

This study has demonstrated a potential connection between resected lung volume, POAF risk, and early-phase non-cancer-related outcomes after lung resection. We have also validated that POAF can be predicted using a published prediction model. If it is technically and oncologically feasible, reducing the volume of resected lung with segmentectomy may help reduce POAF risk and improve non-cancer-related outcomes. The prediction of POAF could also lead to improved postoperative patient care and subsequent outcomes after lung resection. Future directions could involve developing a risk assessment tool incorporating resected lung volume and POAF for perioperative management of high-risk patients, which could provide valuable guidance for treatment decisions and improve patient care.

## Supplementary Material

ivad199_Supplementary_Data

## Data Availability

The data underlying this article will be shared on reasonable request to the corresponding author.

## Notes

Presented at the 60th Annual Meeting of Japan Society of Clinical Oncology, Kobe, Japan, 21 October 2022.

## ABBREVIATIONS

BNP: Brain natriuretic peptide
POAF: Postoperative atrial fibrillation
