# Supplementary material for: Predicting 1-year non-cancer-related adverse events after lung resection
Source: Interdiscip Cardiovasc Thorac Surg. 2023 Dec 12;37(6):ivad199. doi: 10.1093/icvts/ivad199 (PMC10748786; doi:10.1093/icvts/ivad199)
Supplement: ivad199_Supplementary_Data [file ivad199_supplementary_data.docx]

**Supplemental Table S1.** A breakdown of 1-year non-cancer-related adverse events

|  |  | **Grade 3 or 4** | **Grade 5** |
| --- | --- | --- | --- |
|  | **1-year non-cancer-related adverse events*** | **n=83** | **n=11** |
|  | Respiratory diseases | n=66 | n=4 |
|  | Pneumothorax or prolonged air leakage | 24 | 0 |
|  | Pneumonia | 14 | 1 |
|  | Pleural effusion | 8 | 0 |
|  | Empyema | 6 | 0 |
|  | AE-IP | 6 | 1 |
|  | Postoperative atelectasis | 4 | 0 |
|  | Lung abscess | 2 | 0 |
|  | Respiratory failure/ARDS | 1 | 2 |
|  | Bronchopleural fistula | 1 | 0 |
|  | Cardiovascular diseases | n=14 | n=3 |
|  | Arrhythmia | 4 | 0 |
|  | Cerebral stroke | 3 | 2 |
|  | Pulmonary thromboembolism | 2 | 0 |
|  | Aortic aneurysm | 2 | 0 |
|  | Acute cardiac infarction | 1 | 0 |
|  | Peripheral arterial disease | 1 | 0 |
|  | Pulmonary hypertension | 1 | 0 |
|  | Acute aortic dissection | 0 | 1 |
|  | Others | n=3 | n=4 |
| *1-year non-cancer related adverse events was defined as any grade ≥3 conditions using Common Terminology Criteria for Adverse Events grading system. AE-IP, acute exacerbation of interstitial pneumonia; ARDS, acute respiratory distress disease. | | | |

**Supplemental Table S2.** Comparison of 1-year non-cancer-related adverse events between <75- and ≥75-year-old patients by grade

|  |  | **<75 years of age** | | **≥75 years of age** | |  |
| --- | --- | --- | --- | --- | --- | --- |
|  | **Non-cancer-related adverse events** | **n=52** | | **n=42** | | ***P* value** |
|  | Grade 5 adverse events | 4 | (8) | 7 | (17) | **.013** |
|  | Grade 3 or 4 adverse events | 48 | (92) | 35 | (83) |  |
|  | Data were shown as number (%). | | | | | |

**Supplemental Table S3.** Demographics of patients > 75 years old and comparison with and without non-cancer-related adverse events within one year after surgery

|  |  |  |  |  | **Non-cancer-related adverse events within one year** | | | |  |
| --- | --- | --- | --- | --- | --- | --- | --- | --- | --- |
|  | **Characteristics** |  | **All cases (n=156)** | | **No (n=114) (73%)*** | | **Yes (n=42) (27%)*** | | ***P* value** |
|  | Age (y) |  | 78 | (76–80) | 78 | (76–81) | 78 | (76–80) | .97 |
|  | Sex | Male | 84 | (54) | 55 | (48) | 29 | (69) | **.021** |
|  |  | Female | 72 | (46) | 59 | (52) | 13 | (31) |  |
|  | BMI (kg/m^2^) |  | 23 | (21–25) | 23 | (21–24) | 23 | (20–26) | .34 |
|  | Smoking history | Never | 81 | (52) | 63 | (55) | 18 | (43) | .30 |
|  |  | Former | 74 | (47) | 50 | (44) | 24 | (57) |  |
|  |  | Current | 1 | (1) | 1 | (1) | 0 | (0) |  |
|  | Hypertension | No | 66 | (42) | 49 | (43) | 17 | (41) | .78 |
|  |  | Yes | 90 | (58) | 65 | (57) | 25 | (59) |  |
|  | Coronary artery disease | No | 140 | (90) | 104 | (91) | 36 | (86) | .31 |
|  |  | Yes | 16 | (10) | 10 | (9) | 6 | (14) |  |
|  | Diabetes mellitus | No | 116 | (74) | 86 | (75) | 30 | (71) | .61 |
|  |  | Yes | 40 | (26) | 28 | (25) | 12 | (29) |  |
|  | History of atrial fibrillation | No | 145 | (93) | 105 | (92) | 40 | (95) | .50 |
|  |  | Yes | 11 | (7) | 9 | (8) | 2 | (5) |  |
|  | Chemotherapy | No | 154 | (99) | 112 | (98) | 42 | (100) | .39 |
|  |  | Yes | 2 | (1) | 2 | (2) | 0 | (0) |  |
|  | BNP (pg/mL) |  | 31 | (17–52) | 31 | (18–52) | 32 | (16–51) | .95 |
|  | %FEV_1_ (%) |  | 102 | (86–115) | 103 | (88–116) | 92 | (85–110) | .085 |
|  | Surgical approach | Thoracotomy | 127 | (81) | 98 | (86) | 29 | (69) | **.016** |
|  |  | MIS | 29 | (19) | 16 | (14) | 13 | (31) |  |
|  | Surgery | Segmentectomy | 45 | (29) | 37 | (33) | 8 | (19) | .10 |
|  |  | Lobectomy | 111 | (71) | 77 | (67) | 34 | (81) |  |
|  | Number of resected subsegments |  | 6 | (4–10) | 6 | (4–10) | 10 | (6–12) | **.023** |
|  | POAF | No | 137 | (88) | 104 | (91) | 33 | (79) | **.032** |
|  |  | Yes | 19 | (12) | 10 | (9) | 9 | (21) |  |
|  | Data were shown as number (%) or median (25–75 percentiles). *Percentages among all 156 patients. BMI, body mass index; BNP, brain natriuretic peptide; %FEV_1_, percent predicted forced expiratory volume in 1 second; MIA, minimally invasive approach; POAF, postoperative atrial fibrillation | | | | | | | | |
|  |  |  |  |  |  |  |  |  |  |
|  |  |  |  |  |  |  |  |  |  |

**Supplemental Table S4.** Logistic regression for predicting non-cancer-related adverse events within one year in elderly patients (≥ 75 years of age, n=156)

|  |  | **Univariable** | | |  | **Multivariable*** | | |
| --- | --- | --- | --- | --- | --- | --- | --- | --- |
|  | **Characteristics** | **OR** | **(95% CI)** | ***P* value** |  | **OR** | **(95% CI)** | ***P* value** |
|  | Age (per 1 year increase) | 0.97 | (0.85–1.01) | .61 |  |  |  |  |
|  | Sex Female (vs. male) | 0.42 | (0.20–0.89) | **.023** |  | 0.41 | (0.19–0.90) | **.027** |
|  | BMI (per 1 kg/m^2^ increase) | 1.06 | (0.94–1.19) | .35 |  |  |  |  |
|  | Smoking history | 1.00 |  | .36 |  |  |  |  |
|  | Never |  |  |  |  |  |  |  |
|  | Former | 1.68 | (0.822–3.43) |  |  |  |  |  |
|  | Current |  |  |  |  |  |  |  |
|  | Hypertension (vs. none) | 1.11 | (0.54–2.28) | .78 |  |  |  |  |
|  | Coronary artery disease (vs. none) | 1.73 | (0.59–5.11) | .32 |  |  |  |  |
|  | Diabetes mellitus (vs. none) | 1.23 | (0.56–2.72) | .61 |  |  |  |  |
|  | History of atrial fibrillation (vs. none) | 0.58 | (0.12–2.82) | .50 |  |  |  |  |
|  | Chemotherapy (vs. none) |  |  | 1.00 |  |  |  |  |
|  | BNP (per 1 pg/mL increase) | 1.00 | (0.99–1.01) | .63 |  |  |  |  |
|  | %FEV_1_ (per 1 % increase) | 0.98 | (0.97–1.00) | .087 |  |  |  |  |
|  | Surgical approach MIS (vs. thoracotomy) | 0.36 | (0.16–0.84) | **.019** |  |  |  |  |
|  | Lobectomy (vs. segmentectomy) | 2.04 | (0.86–4.85) | .11 |  |  |  |  |
|  | Number of resected subsegments (per 1 increase) | 1.13 | (1.02–1.25) | **.025** |  | 1.13 | (1.01–1.26) | **.035** |
|  | POAF (vs. none) | 2.84 | (1.06–7.57) | **.037** |  | 2.84 | (1.02–7.88) | **.045** |
|  | *Multivariable analysis using a backward selection strategy, starting with factors with P a value of ≤ 0.1 in univariable analysis. BMI, body mass index; BNP, brain natriuretic peptide; CI, confidence interval; %FEV_1_, percent predicted forced expiratory volume in 1 second; MIS, minimally invasive surgery; OR, odds ratio; POAF, postoperative atrial fibrillation. | | | | | | | |
|  |  |  |  |  |  |  |  |  |
|  |  |  |  |  |  |  |  |  |
